# Metformin-Induced Generalized Bullous Fixed-Drug Eruption with a Positive Dechallenge-Rechallenge Test: A Case Report and Literature Review

**DOI:** 10.1155/2023/6353919

**Published:** 2023-03-31

**Authors:** Bahareh Abtahi-Naeini, Tooba Momen, Rezvan Amiri, Parvin Rajabi, Fereshte Rastegarnasab

**Affiliations:** ^1^Pediatric Dermatology Division of Department of Pediatrics, Imam Hossein Children's Hospital, Isfahan University of Medical Sciences, Isfahan, Iran; ^2^Skin Diseases and Leishmaniasis Research Center, Isfahan University of Medical Sciences, Isfahan, Iran; ^3^Department of Asthma, Allergy and Clinical Immunology, Child Growth and Development Research Center, Research Institute of Primordial Prevention of Non-Communicable Disease, Isfahan University of Medical Sciences, Isfahan, Iran; ^4^Leishmaniasis Research Center, Kerman University of Medical Sciences, Kerman, Iran; ^5^Department of Pathology, School of Medicine, Isfahan University of Medical Sciences, Isfahan, Iran; ^6^Student Research Committee, Isfahan University of Medical Sciences, Isfahan, Iran

## Abstract

Metformin is a commonly used medication in diabetic patients. It can cause different complications including cutaneous adverse reactions. Metformin-induced fixed-drug eruption (FDE) has been reported in limited cases. Due to the popularity of metformin, clinicians need to be aware of uncommon drug reactions for proper diagnosis and treatment. Herein, we report a 43-year-old man with generalized bullous lesions with a positive dechallenge-rechallenge test diagnosed as metformin-induced generalized bullous fixed-drug eruption. Metformin dosage was stopped and lesions were treated with topical clobetasol propionate and oral prednisolone and cyclosporine-A. After a 6-month follow-up, he was well without any relapsing episodes.

## 1. Introduction

Metformin is a common orally administered drug mainly used for the treatment of diabetes mellitus (DM) type 2 [1]. In addition to the control of glucose metabolism, metformin can be used for other therapeutic options such as anticancer, antiaging, protection of cardiovascular and neurologic systems, or treating polycystic ovary syndrome [2]. It has been reported as a cost-effective drug for reducing weight in obese patients [3].

The most prevalent complication of metformin is gastrointestinal manifestations, such as nausea, vomiting, and diarrhea [4]. Metformin-associated lactic acidosis is a rare adverse effect mostly in patients with underlying conditions that can lead to severe dramatic symptoms such as complete transient blindness [5]. Rarely metformin can also trigger cutaneous adverse drug reaction (CADR)-like leukocytoclastic vasculitis [6], DRESS syndrome [7], psoriasiform and lichenoid drug eruption [8], photosensitivity reactions [9], and fixed-drug eruption (FDE) [10, 11].

Generalized bullous FDE is a rare variant of FDE, presented as classic FDE lesions involving at least 10% of the body surface area with superimposed bullae [12].

To date, six other cases have been reported with metformin-induced FDE.

Since metformin is a commonly used medication, clinicians must be aware of the unusual manifestations of this drug to provide proper diagnosis and treatment, on-time discontinuation of the drug, and avoid unnecessary further evaluation.

Herein, we report a 43-year-old man with generalized bullous lesions with a positive dechallenge-rechallenge test diagnosed as metformin-induced generalized bullous FDE.

## 2. Case Presentation

A 43-year-old man was referred with complaints of recurrent episodes of generalized bullous lesions distributed at the anterior and posterior trunk, upper and lower extremities, and genital and labial mucosa. He had started metformin about 5 years ago for losing weight. He did not have any history of DM, hypertension, or any other underlying disease. He used metformin in an irregular and intermittent pattern. He did not use any herbal extract or other medications.

The first time, lesions showed up two weeks after starting metformin. Lesions continued to happen in an on and off pattern with a generalized distribution.

**Figure 1:**
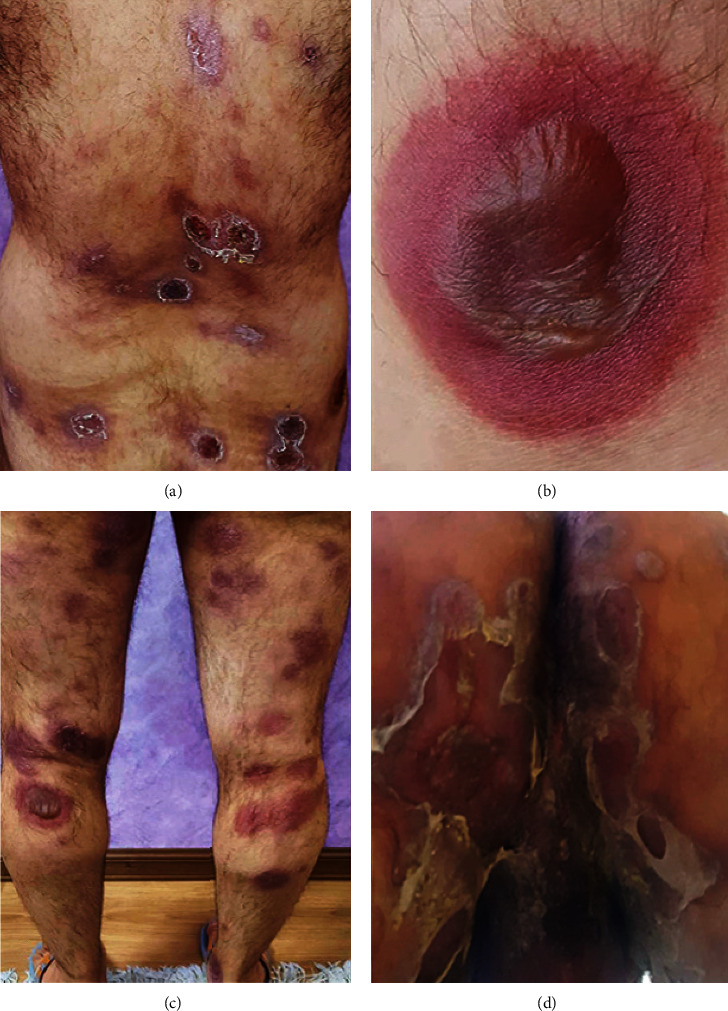
Generalized bullous fixed-drug eruption. (a, b) A positive rechallenge phenomena and reappearing of generalized bullous formation after restarting of metformin. (c, d) Bullous formation with central dusky appearance after restarting of metformin on the lower extremities and perineum.

Cutaneous examination revealed multiple well-defined erythematous to violaceous plaques with a central dusky appearance in association with bulla formation over the chest, extremities, genitalia, and lips (Figures 1(a) and 1(b)).

**Figure 2:**
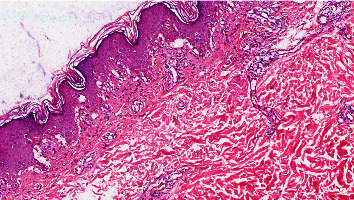
Histopathology of fixed-drug eruption associated with metformin. The histopathologic feature shows vacuolar interface dermatitis, necrotic keratinocytes, and eosinophil infiltration.

Due to the widespread skin denudation with clinical suspicion of generalized bullous FDE, recurrent Stevens–Johnson syndrome/toxic epidermal necrolysis (SJS/TEN), and bullous pemphigoid, a punch biopsy was taken. Histopathology demonstrated vacuolar interface dermatitis, apoptotic keratinocytes, and pigment incontinence associated with eosinophil infiltration that were consistent with bullous FDE (Figure 2).

The causality assessment was carried out using the Naranjo ADR Probability Scale. Our patient's total Naranjo Scale score was 10 as a definite adverse drug reaction [13] (Table 1).

**Figure 3:**
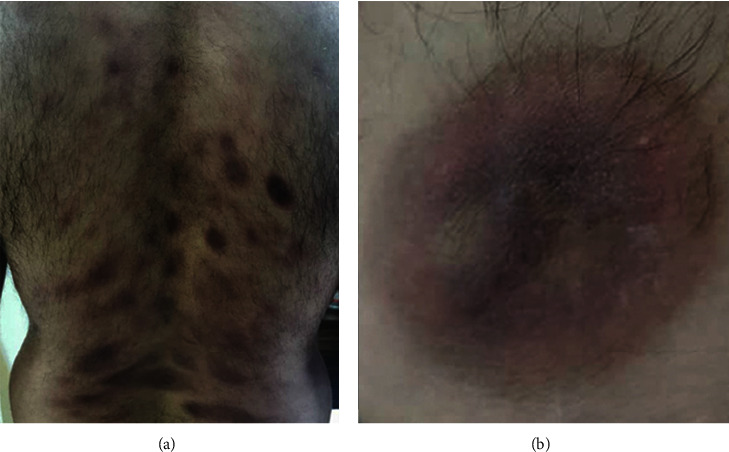
Postinflammatory hyperpigmentation in generalized fixed-drug eruption. A positive dechallenge phenomenon and subsiding of the bullous formation after the discontinuation of the metformin.

A diagnosis of metformin-induced generalized bullous FDE was made, and the patient was told to stop the offending agent. After discontinuation of metformin, the progression of the disease was stopped (Figure 3) and the patient was treated with oral prednisolone (40 mg daily) and cyclosporine-A (100 mg daily). During the treatment course, the patient arbitrarily decided to rechallenge the drug. At the time he restarted the metformin, lesions became more pronounced and more severe. They flared up and widespread skin denudation and bullous lesions occurred with erosions and ulcers compatible with a positive rechallenge test to the metformin (Figures 1(c) and 1(d)).

Metformin again was stopped and lesions were treated with topical clobetasol propionate and oral prednisolone (60 mg daily) and cyclosporine-A (150 mg daily) and gradually tapered after clinical improvement. After a 6-month follow-up, he was well without any relapsing episodes.

## 3. Discussion

This is the first report of metformin-induced generalized bullous FDE with a positive dechallenge-rechallenge test in a nondiabetic patient. Presentation of generalized FDE in diabetic patients as a CADR is extremely rare. The point that makes our case very impressive is the generalization of the bullous lesions and the long lag period for the true diagnosis.

The most frequent drugs triggering FDE included antibiotics (e.g., trimethoprim-sulfamethoxazole, tetracycline, penicillin, and quinolones), nonsteroidal anti-inflammatory drugs (NSAIDs), hypnotics (e.g., barbiturates), and anticonvulsants (e.g., carbamazepine), but potentially the list of the drug is open [10, 14].

Usually, FDE manifested as a cutaneous immunological reaction in which lesions can reappear at the same site, after re-exposure to the causative drug. Lesions can involve the lips, palms of the hands, the lower back, the hip, groin areas, and soles of the feet [10, 14]. The lesions usually appear as solitary, erythematous macules that can progress to edematous plaques or bullous-type lesions [15].

Generalized bullous fixed-drug eruptions (FDEs) are defined as blisters and erosions involving at least ten percent of the whole-body surface area and at least three of six anatomical sites [12]. An important differential diagnosis is SJS/TEN based on the widespread distribution, dusky coloration, and skin detachment [15].

Metformin-induced FDE has been reported in six cases [10, 11, 14, 16–18] (Table 2). All of these cases are more than 40 years of age; 3 of them are female and 3 are male. The lower limb is the most prevalent involved location (5 cases) [10, 11, 14, 17, 18]. Only one of the patients had lesions on her face [16]. There is no report of FDE lesions involving the trunk, but it was seen in our case for the first time. All of the cases had a multiple drug regimen [10, 11, 14, 16–18], but in our case the only responsible drug is metformin.

The Naranjo Scale is reported in three of the cases and ranges 5–8, which shows a probable adverse drug reaction [11, 14, 17]. It was measured ten in our case, which is compatible with a definite adverse drug reaction (ADR) due to metformin (Table 1).

Discontinuation of metformin was the main strategy in all patients [10, 11, 14, 16–18], as carried out in our case. Oral corticosteroids had no positive effect [11, 16, 17].

The treatment of FDE is mainly recognizing and discontinuation of the responsible drug. Also, systemic antihistamines and topical or systemic corticosteroids can be used in severe and symptomatic patients [10].

Due to its rarity, optimal treatment of generalized bullous FDE is not yet been established. Current management includes immediate cessation of the offending drug and supportive therapy with antihistamines, analgesics, or antiseptics. The efficacy of steroids is not proven but they are used in severe conditions [15]. In our case, metformin dosage was stopped and lesions were treated with topical clobetasol propionate, oral prednisolone, and cyclosporin.

## 4. Conclusion

Because of the potential recurrence of FDE and the clinical importance of generalized bullous FDE related to metformin, the current report provides information about a new case of generalized bullous FDE in association with metformin.

## Figures and Tables

**Table 1:** The Naranjo algorithm for adverse drug reaction for a patient with metformin-induced fixed-drug eruption.

Questions	Yes	No	Do not know	Score
(1) Are there previous conclusive reports on this reaction?	+1	0	0	+1
(2) Did the adverse event appear after the suspected drug was administered?	+2	−1	0	+2
(3) Did the adverse event improve when the drug was discontinued or a specific antagonist was administered?	+1	0	0	+1
(4) Did the adverse event reappear when the drug was readministered?	+2	−1	0	+2
(5) Are there alternative causes that could on their own have caused the reaction?	−1	+2	0	+2
(6) Did the reaction reappear when a placebo was given?	−1	+1	0	0 (do not know)
(7) Was the drug detected in blood or other fluids in concentrations known to be toxic?	+1	0	0	0 (do not know)
(8) Was the reaction more severe when the dose was increased or less severe when the dose was decreased?	+1	0	0	0 (do not know)
(9) Did the patient have a similar reaction to the same or similar drugs in any previous exposure?	+1	0	0	+1
(10) Was the adverse event confirmed by any objective evidence?	+1	0	0	+1
	Total score: 10			

*Note.* Naranjo Adverse Drug Reaction Probability Scale: ≥9 = definite adverse drug reaction; 5–8 = probable adverse drug reaction; 1–4 = possible adverse drug reaction; 0 = doubtful adverse drug reaction.

**Table 2:** Reported cases of metformin-induced fixed-drug eruption.

No.	Author/year	Age (year old)/sex	Dermatologic manifestation	Histopathology	Naranjo scale [13]	Treatment
1	Monroe/2010 [16]	41/female	Round, purplish-brown, targetoid macules ranged in size from 1 to 3 cm on the lips, face, and arms and some edematous lesions	Interface changes, scattered necrotic keratinocytes, and epidermal pigmentary incontinence	NA^*∗*^	Discontinuation of metformin oral antibiotics, corticosteroids, and acyclovir had no effect
2	Steber et al./2016 [14]	56/female	Small, round, erythematic, pustular lesions on palms and soles	NA	Naranjo = 8	Discontinuation of metformin
3	Ramirez Bellver et al./2017 [11]	86/male	Round/oval, erythematous pruritic macules, and patches, located on his lower limbs mostly on his buttocks, forearms, and hands	Cutaneous hemophagocytosis/superficial and deep perivascular dermal infiltrate interface dermatitis and necrotic keratinocytes/necrotic keratinocytes concentrated at the acrosyringium/deep dermal infiltrate composed of histiocytes	Naranjo = 5	Discontinuation of metformin oral prednisone for 2 weeks had no effect
4	Sharma et al./2017 [17]	47/male	Round/oval erythematous macules, tender palpable purpura patches on the lower limbs, lower back, and buttocks	NA	Naranjo = 6	Discontinuation of metformin oral prednisone cream of synthetic glucocorticoid (fluticasone) had no effect
5	Togawa et al./2019 [18]	46/male	Skin rashes on the lower left thigh and the back of the body after taking metformin and also after metformin hydrochloride TE	NA	NA	Discontinuation of inactive ingredients of metformin
6	Al Masri et al./2021 [10]	58/female	Itching and burning sensation over the right leg accompanied by the appearance of blisters, ulcerations, and erythema after starting metformin and also after gliclazide, vildagliptin, empagliflozin, and liraglutide	Lichenoid drug eruption	NA	Discontinuation of metformin
7	Our case	43/male	Multiple well-defined erythematous to violaceous plaques with a central dusky appearance in association with bulla formation over the chest, extremities, genitalia, and lips	Vacuolar interface dermatitis, apoptotic keratinocytes, and pigment incontinence associated with eosinophils infiltration	Naranjo = 10	Discontinuation of metformin

^*∗*^NA = Not available.

## Data Availability

The data used to support the findings of this study are available from the corresponding author upon request.
